# Hepatic actinomycosis mimicking an isolated tumor recurrence

**DOI:** 10.1186/1477-7819-9-70

**Published:** 2011-07-11

**Authors:** Michael G Wayne, Rahul Narang, Arif Chauhdry, Justin Steele

**Affiliations:** 1 Pancreas Center at Beth Israel Medical Center, NY 37 Union Square West, NY 10003, USA

## Abstract

Actinomyces species has been described as an opportunistic pathogen, particularly in the oral cavity; however, in rare cases these bacteria can cause actinomycosis which is characterized by formation of abscesses in the mouth, lungs, or gastrointestinal tract.

Actinomycosis was commonly present in the pre-antibiotic era; however, it has a low prevalence now days. It has been recognized since 150 years ago, but because of its variable clinical presentation and indolent course, its recognition is difficult and patients are often misdiagnosed. Here we present a case of primary hepatic actinomycosis presenting as a metastatic liver tumor.

## Case report

This is the case of a 65-year-old male, who originally presented on August 18, 2008 with obstructive jaundice. His past medical history includes diabetes and hypertension. He underwent ERCP with stenting of the bile duct. The patient also had a spiral CT and an endoscopic ultrasound of the pancreas. These tests helped to determine resectability. He underwent a pancreaticoduodenectomy (Whipple procedure) on Sept. 8, 2008 for a pancreatic head adenocarcinoma. He had a standard whipple performed, with removal of the gallbladder and distal stomach, as well as the head of the pancreas and duodenum. There were no intraoperative events and no gallstones were spilled. He received adjuvant chemotherapy for 7 months. The chemotherapy was gemzar, oxaliplatinum and tarceva. The patient had routine follow-up and surveillance for recurrence every 4 months the first year and every 6 months for the second year. This included a CT scan and CA 19-9 level. Over the course of one year he was admitted to the hospital on several occasions for low-grade fever, for which the diagnosis was not established, and at times treated as an outpatient for multiple urinary tract infections. During these episodes the patient always had a normal white blood cell count and normal neutrophils/lymphocytes on differential. At no time did he complain of any abdominal pain or any difficulty tolerating food. All CT scans during this first year were negative for recurrent malignant disease.

**Figure 1:**
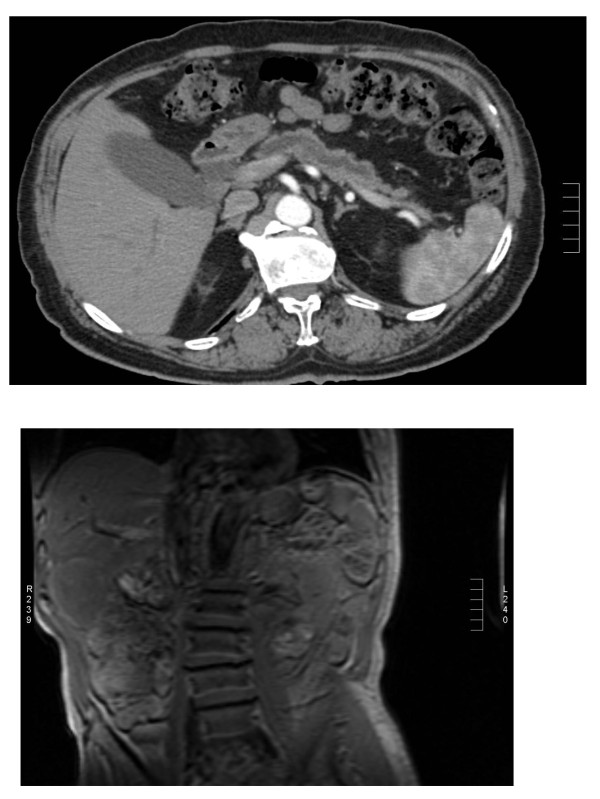
**Liver lesion present in the inferior aspect of segment 6 (arrow)**. This lesion appears to be an isolated tumor recurrence 18 months after Whipple procedure.

**Figure 2:**
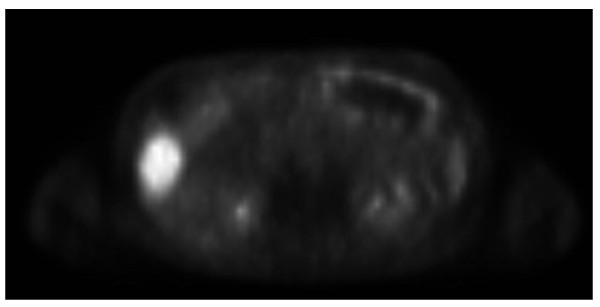
**PET scan showed abnormal robust focus of metabolic activity in inferior lateral aspect of right hepatic lobe concerning for metastatic disease (arrow)**.

At approximately 18 months in follow-up, the repeated computed tomography (CT) scan showed a non-specific abnormality in the right lobe of the liver, suspicious for a mass. A magnetic resonance imaging (MRI) and positron emission tomography (PET) scan were then performed, which was positive for a mass, suspicious for an isolated tumor recurrence between segments 5 and 6 in the liver. Both the CT and MRI showed a large liver lesion present in the inferior aspect of segment 6 measuring 7 cm AP, 5 cm transverse, and 4.5 cm craniocaudal (Figure 1). This mass demonstrated heterogeneous thick peripheral rim enhancement with central hypo-enhancement and likely necrosis. The PET scan showed an abnormal robust focus of metabolic activity in inferior lateral right hepatic lobe, concerning for metastatic disease, SUV 11.6 (Figure 2).

Laboratory examination of the patient's blood demonstrated: white blood cell count 6.1 (reference range 4.5-10.8 K/uL), alkaline phosphatase level 141 (reference range 38-126 U/L), and AST 36 (reference range 15-46 U/L), ALT 50 (reference range 13-69 U/L). The total and direct bilirubin levels were normal. The CA 19-9 was 84, which was increased from 39 on the previous sample from 3 months prior. Physical exam of the abdomen was unremarkable. No masses were palpated, the liver was not enlarged, and the abdomen was non-tender. There was no cervical, umbilical, or inguinal nodes appreciated.

FNA biopsy was discussed at the GI tumor board, but in the setting of a rising CA19-9 and suspicious CT, MRI, and PET scan, the decision was made to proceed to surgery without a biopsy. The tumor board felt that this was recurrent pancreatic cancer and because of this decided the small risk of seeding the needle tract by doing an FNA was not warranted in this case.

Patient underwent a resection of segment 5 and 6 of the liver without any complications. Final pathology of the specimen returned as inflammatory granulation tissue and granules consistent with hepatic actinomycosis. This was confirmed on tissue cultures. Special staining of this specimen showed gram positive filamentous bacteria.

Infectious disease consult was called to review the case. They selected doxcycline iv, based on culture and sensitivity report, which would be changed to po doxcycline on discharge. The patient had an uneventful recovery and was discharged home on post-operative day 5. He was discharged home on oral doxycycline for a 6 month treatment course. 30-day follow-up shows no recurrence of this lesion and he continues to remain afebrile.

## Discussion

The genus Actinomyces species are a slowing growing, gram-positive, non-spore-forming bacteria, that thrive in microareophilic and anaerobic conditions [1]. There are 13 different species of actinomycosis of which 6 are associated with human disease (arachina propionica, bovis, israelli, naseslundii, odontolyticus and viscosus). The most common pathogen encountered is *Actinomyces israelii*, which gives rise to chronic suppurative infection [2,3].

They tend to be associated with infections of the cervicofacial and oral region. A rare cause of abdominal infection, the pathogenesis is presumed to be caused by hematogeneous spread via the portal vein from either a mucosal injury or other abdominal focus of injury and/or infection. The non-specific symptoms of fever, weight loss and abdominal pain make diagnosis of this condition a challenge. Despite advanced imaging techniques and difficulty in obtaining in positive cultures, the rate of preoperative diagnosis is less than 10% [4].

According to the literature, actinomycosis of the abdomen and pelvis accounts for 10-20% of reported cases. Typically, these patients have a history of recent or remote bowel surgery (eg, perforated appendicitis, perforated colonic diverticulitis, spilled gallstones during cholecystectomy) or ingestion of foreign bodies (eg, chicken or fish bones), during which actinomycetes are introduced into the deep tissues. Diagnosis is usually established postoperatively, following exploratory laparotomy for a suspected malignancy. Pelvic actinomycosis most commonly ascends from the uterus in association with intrauterine contraceptive devices (IUCDs). Female genital *Actinomyces *infection is relatively rare, although strongly related to long-lasting intrauterine contraceptive device (IUD) application. An infective pathway has been postulated extending upward from the female perineum to the vagina and cervix. The traumatic effect of the device and a prior infection may contribute to the *Actinomyces *infection in the female genitalia.

Risk factors associated with this condition include abdominal wall trauma, gastrointestinal foreign body, previous abdominal/pelvic surgery, gastrointestinal tract lesions and immunosuppression [5]. Since gastrointestinal foreign bodies are a risk factor it is of no surprise that recent literature has cited an increased risk for this infection with biliary and pancreatic stent placement [6]

Liver involvement has been reported in 15% of the cases, with majority of them resulting from translocation from either an intra-abdominal or intrathoracic site [7]. Primary liver involvement has only been reported in 5% of the cases [7].

Primary hepatic actinomycosis typically presents with an indolent course with symptoms typically present for greater than two weeks [8]. Immunocomptetent adults with variable age distribution and a male prevalence has been observed [8].

Difficult task for the management of actinomycosis is to reach the diagnosis before surgical approach is taken. In our patient the patient presents with isolated hepatic mass on MRI and PET scan strongly suspicious for recurrence of tumor after one year of tumor free survival. Because imaging studies frequently reveal single or multiple lesions, actinomycosis is often misdiagnosed as a primary or metastatic tumor. Radiographicallly, lesions frequently present as a single hypodense mass/abscess 68.4%) [9].

For diagnosis, macroscopic, microscopic and histochemical examinations of surgical specimens are required, but the definitive diagnosis is based on tissue culture. The infection tends to lead a chronic and suppurative infection leading to fibrosis with draining sinuses that are pale yellow and often referred to as "sulfur granules" [6]. The wall of these mass are often "wooden" in consistency [2]. Our patient presented with one year history of indolent course with nonspecific and undiagnosed causes of fevers and hospital admissions, treated with antibiotics. He never had an immunosuppressive work-up but his white blood cell count and total lymphocyte count were always normal.

Treatment of this infection consists of intravenous penicillin-G for four weeks and then oral penicillin-V for 12 months [10,11]. Prolonged treatment is recommended because of the incidence of recurrence. Although no true surgical intervention guidelines has been established, it has been used for treatment with patients who present with extensive necrotic tissue or large abscesses that cannot be adequately drained [12,13]. Our patient's actinomycoses was sensitive to both penicillin-G and doxcycline. Doxycycline was the antibiotic selected by our infectious disease doctor. No specific reason given for his choice.

## Conclusions

Our conclusion from this case report is that primary hepatic actinomycosis can present in patients as a solitary hepatic mass after gastrointestinal malignancy. In our case it was mimicking a recurrence of the tumor. It is a rare and often overlooked etiology for a liver mass. An indolent course with unexplainable fevers, occasionally requiring hospital admissions, in patients with history of GI malignancies, should make physicians aware of this possible diagnosis. Nevertheless, the variable clinical presentation of this disease and its low incidence makes it difficult to recognize.

## Consent

Written informed consent was obtained from the patient for publication of this Case report and any accompanying images. A copy of the written consent is available for review by the Editor-in-Chief of this journal

## Competing interests

The authors declare that they have no competing interests.

## Authors' contributions

MW-main author and primary surgeon for patient; RN-assisted in writing case report; AC-assisted in gathering data; JS-assistant surgeon and helped edit the article; All authors read and approved the final manuscript.

## References

[B1] Bowden GHW Baron S et al. Actinomycosis in:Baron's Medical Microbiology Univ of Texas Medical Branch 1996 4

[B2] Brook I Actinomycosis: diagnosis and management South Med J 2008 101 1019 23 10.1097/SMJ.0b013e3181864c1f 18791528

[B3] Jermini I An unusual case of hepatic abscess Praxis 1994 20 1781 4 10.1024/0369-8394.93.43.1781 15553899

[B4] Brown JR Human actinomycosis: CT features J Comput Assist Tomogr 1986 10 335 337 10.1097/00004728-198603000-00035 3512640

[B5] Lee JD Kim PG Jo HJ A case of primary hepatic Actinomycosis J Korean Med Sci 1993 8 385 9 8305147 10.3346/jkms.1993.8.5.385 PMC3053711

[B6] Kanellopoulou T Primary Hepatic Actinomycosis American J Med Sci 2010 4 362 65 10.1097/MAJ.0b013e3181cbf47c 20195148

[B7] Gulglielmi A Primary hepatic actinomycosis: a clinical case report and review of literature Ann Ital Chir 1991 62 185 9 1755599

[B8] Lai AT Hepatic actinomycosis presenting as a liver tumor: a case report and literature review Asian J Surg 2004 27 345 7 10.1016/S1015-9584(09)60066-X 15564194

[B9] Christodoulou N Papadakis I Velegrakis M Actinomycotic live abscess. Case report and review of the literature Chir Ital 2004 56 1 141 146 15038660

[B10] Felekouras E Menenakos C Griniatsos J Deladetsima I Kalaxanisi N Nikiteas N Papalambros E Kordossis T Bastounis E Liver resection in cases of isolated hepatic actinomycosis: case report and review of the literature Scand J Infect Dis 2004 36 6-7 535 538 10.1080/00365540410020866-1 15307597

[B11] Chen LW Chang LC Shie SS CHien RN Solitary actinomycotic abscesses of the liver: report of two cases Int J Clin Pract 2006 60 1 104 107 16409437 10.1111/j.1368-5031.2005.00691.x

[B12] Islam T Athar MN Athar MK Usman MH Misbah B Hepatic actinomycosis with infiltration of the diaphragm and right lung: a case report Can Respir J 2005 12 6 336 337 16247532 10.1155/2005/804093

[B13] Lall T Isolated hepatic actinomycosis: A case report J Med Case Report 2010 4 45 10.1186/1752-1947-4-45 PMC2833155 20181118

